# Comparison of oncological benefits of deep neuromuscular block in obese patients with gastric cancer (DEBLOQS_GC study): A study protocol for a double-blind, randomized controlled trial

**DOI:** 10.1097/MD.0000000000013424

**Published:** 2018-12-10

**Authors:** Yoontaek Lee, Donghwan Ha, Liang An, You-Jin Jang, Hyub Huh, Chang Min Lee, Yeon-Hee Kim, Jong-Han Kim, Seong-Heum Park, Young-Jae Mok, Il Ok Lee, Oh Kyoung Kwon, Kyung Hwa Kwak, Jae Seok Min, Eun Jin Kim, Sung Il Choi, Jae Woo Yi, Oh Jeong, Mi Ran Jung, Hong Bum Bae, Joong-Min Park, Yong Hoon Jung, Jin-Jo Kim, Dal Ah Kim, Sungsoo Park

**Affiliations:** a Department of Surgery; b Department of Anesthesiology and Pain Medicine, Korea University College of Medicine, Seoul; c Department of Surgery; d Department of Anesthesiology and Pain Medicine, Kyungpook National University Medical Center, Daegu; e Department of Surgery; f Department of Anesthesiology and Pain Medicine, Dongnam institute of Radiological & Medical Sciences, Cancer Center, Busan; g Department of Surgery; h Department of Anesthesiology and Pain Medicine, Kyung Hee University Hospital at Gangdong, Seoul; i Department of Surgery; j Department of Anesthesiology and Pain Medicine, Chonnam National University Hwasoon Hospital, Hwasun; k Department of Surgery; l Department of Anesthesiology and Pain Medicine, Chung-Ang University College of Medicine; m Department of Surgery; n Department of Anesthesiology and Pain Medicine, Incheon St. Mary's Hospital, The Catholic University of Korea College of Medicine, Incheon, Korea.

**Keywords:** gastric neoplasm, laparoscopy, lymphadenectomy, neuromuscular block

## Abstract

**Purpose:**

Many studies have demonstrated the advantage of maintaining intraoperative deep neuromuscular block (NMB) with sugammadex. This trial is designed to evaluate the impact of muscle relaxation during laparoscopic subtotal gastrectomy on the oncological benefits, particularly in obese patients with gastric cancer.

**Materials and methods:**

This is a double-blind, randomized controlled multicenter prospective trial. Patients with clinical stage I–II gastric cancer with a body mass index of 25 and over, who undergo laparoscopic subtotal gastrectomy will be eligible for trial inclusion. The patients will be randomized into a deep NMB group or a moderate NMB group with a 1:1 ratio. A total of 196 patients (98 per group) are required. The primary endpoint is the number of harvested lymph nodes, which is a critical index of the quality of surgery in gastric cancer treatment. The secondary endpoints are surgeon's surgical condition score, patient's sedation score, and surgical outcomes including peak inspiratory pressure, operation time, postoperative pain, and morbidity.

**Discussion:**

This is the first study that compares deep NMB with moderate NMB during laparoscopic gastrectomy in obese patients with gastric cancer. We hope to show the oncologic benefits of deep NMB compared with moderate NMB during subtotal gastrectomy.

**Trial registration number:**

ClinicalTrials.gov (NCT03196791), date of registration: October 10, 2017.

## 1 Introduction

Gastric cancer is the third most common cancer and one of the leading causes of cancer death worldwide.^[1]^ The standard surgical procedures for treating gastric cancer include gastrectomy and lymphadenectomy, and several studies have shown that the retrieval of higher lymph node yields correlate with improved survival for stage-specific gastric cancer.^[2,3]^ In obese patients, high body mass index (BMI) has been associated with increased operative time, blood loss, and complications,^[4]^ resulting in inadequate lymphadenectomy in patients with high BMI. In the literature, high BMI has been considered a contraindication to laparoscopic surgery because of technical difficulties, especially in the case of lymph node dissections, and of high conversion rates owing to difficult lymph node dissection or BMI-related intraoperative complications.^[5,6]^ However, as experience with laparoscopic methods has accumulated, recent studies have reported the feasibility of laparoscopic gastrectomy in obese patients.^[7,8]^

Although deep neuromuscular block (NMB) offers advantages in terms of improving surgical conditions and reducing intra-abdominal pressure during laparoscopic surgery, it has been avoided owing to concerns regarding recovery at the end of the operation. However, with the advent of sugammadex (Bridion, Merck Sharp and Dohme, Oss, The Netherlands), immediate reversal of deep NMB became feasible without residual blockage.^[9,10]^ Several studies reported that deep NMB could improve surgical conditions as compared to moderate NMB during laparoscopic surgery (hysterectomy, cholecystectomy, colectomy, etc.).^[11–15]^

These previous studies, however, mainly measured subjective parameters, and did not explore the potential therapeutic effect of deep NMB during laparoscopic surgery. To evaluate the surgical benefits of deep NMB, studies focusing on technically demanding procedures including laparoscopic lymphadenectomy on patients with high BMI are necessary. To date, there have been no studies performing during laparoscopic gastrectomy on patients with gastric cancer and evaluating the oncological benefits of deep NMB. Therefore, we propose this double-blind, randomized controlled trial to evaluate the impact of muscle relaxation during laparoscopic gastrectomy on obese patients with gastric cancer.

## 2 Materials and methods

### 2.1 Objectives

The study objective is to demonstrate the efficacy of deep NMB in obese patients with clinical stage I–II gastric cancer who undergo laparoscopic subtotal gastrectomy. The primary endpoint is the number of harvested lymph nodes, which is a critical index of the quality of gastric cancer surgery. The secondary endpoints are surgeon's surgical condition score, patient's sedation score, and surgical outcomes including peak inspiratory pressure, operation time, postoperative pain, and morbidity.

### 2.2 Study design

This is a randomized controlled trial comparing deep NMB with moderate NMB in laparoscopic subtotal gastrectomy in obese patients with gastric cancer. The design of the study is blinded (the surgical team and the research team are all blinded to the treatment); the attending anesthesiologist is not blinded.

This study was approved by the institutional review board (IRB) of each participating institution. All participating investigators obey the guidelines of the Declaration of Helsinki.^[16]^

### 2.3 Study population

The patient inclusion criteria are as follows:

(1) scheduled for laparoscopic distal gastrectomy with D1 + or D2 lymphadenectomy (lymphadenectomy is performed on the basis of the criteria of the Japanese Gastric Cancer Treatment Guidelines 2014 ^[17]^) with histologically proven gastric adenocarcinoma;
(2) age between 19 and 75 years;
(3) clinical stage I–II gastric cancer according to the 8th edition of the American Joint Committee on Cancer System ^[18]^ (clinical stage is determined based on gastrofiberscopy and abdominal computed tomography [CT] findings);
(4) BMI >25 kg/m^2^; and
(5) American Society of Anesthesiologists Physical status I or II.

The patient exclusion criteria are the following:

(1) family history of malignant hyperthermia;
(2) significant renal or hepatic dysfunction, known or suspected neuromuscular disorders, allergies to narcotics or muscle relaxants, known previous abdominal surgeries; and
(3) receiving medication known to interfere with neuromuscular function (e.g., aminoglycosides, anticonvulsants, or magnesium).
(4) All patients indicate willingness to participate in this study by providing informed consent and can decide to withdraw from the study at any time.

### 2.4 Study protocol

**Figure 1:**
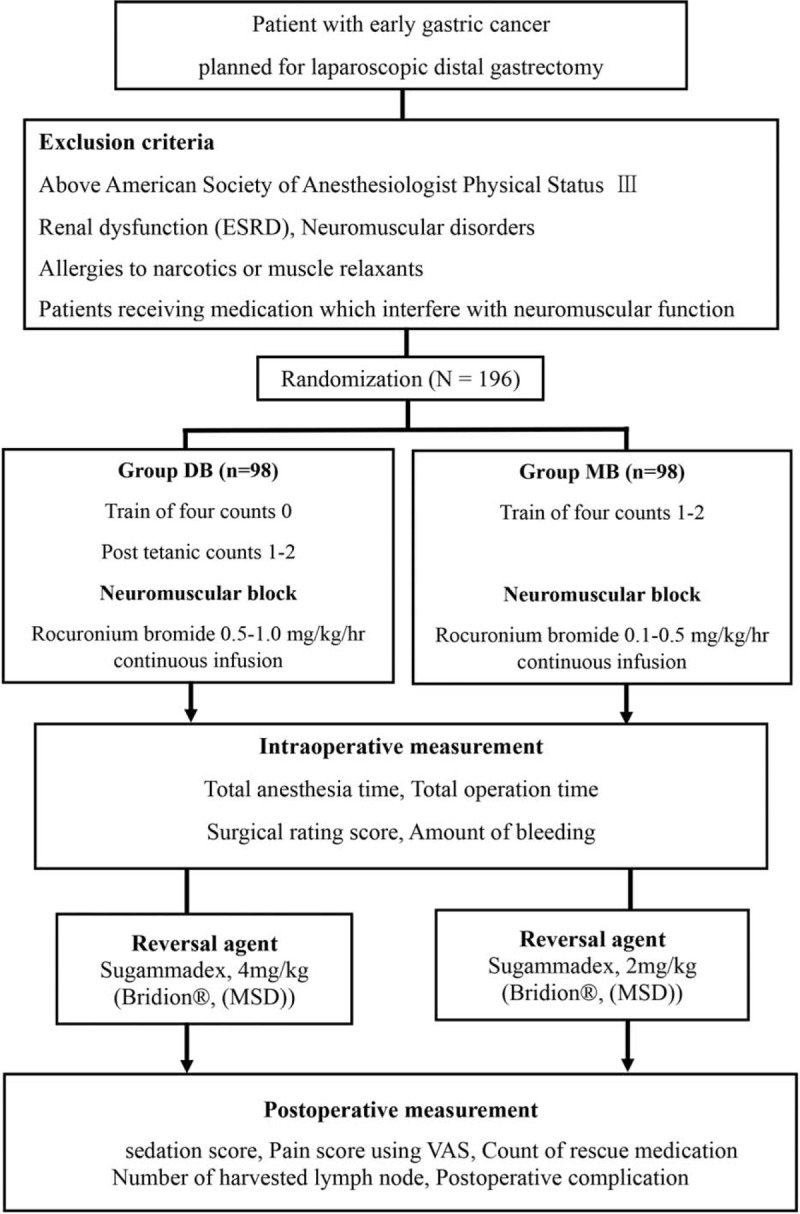
Overview of the DEBLOQS_GC RCT design. ESRD = end-stage renal disease, DB = deep block, MB = moderate block, RCT = randomized controlled trial.

The Korea University Medical Center Anam Hospital will manage this clinical trial. The patients are assigned to the deep NMB group or the moderate NMB group. The clinical research coordinator (CRC) uses a computer randomization program to determine the degree of muscle relaxation of each patient. Stratified block randomization is used to guarantee even distribution, which means that all participating hospitals are assigned moderate or deep NMB randomly in a 1:1 ratio. The CRC notifies the anesthesiologist of the degree of muscle relaxation just before the operation. Surgeons are blinded to the degree of muscle relaxation: They can enter the operating room after the muscle relaxation has been performed. A train-of-four (TOF) monitor is placed out of the surgeon's visual field. The anesthesiologist injects sugammadex after confirming the absence of the surgeon. The ward staff members who evaluate patient outcomes are blinded to the degree of muscle relaxation. The overall flow chart is shown in Figure 1.

### 2.5 Anesthesia procedure

Standard monitoring including electrocardiogram, noninvasive blood pressure, and pulse oximetry are done in the operation room. Anesthesia is induced and maintained with propofol and remifentanil using total intravenous anesthesia-target controlled infusion (TIVA-TCI). Following confirmation of loss of consciousness, neuromuscular activity monitoring via acceleromyography (TOF-Watch SX; Organon; Roseland, NJ) is initiated at the adductor pollicis muscle. Neuromuscular monitoring is performed in accordance with the Good Clinical Research Practice guidelines.^[19]^ Before rocuronium administration, the TOF-Watch SX is calibrated and stabilized. The ulnar nerve is stimulated supramaximally near the wrist with square pulses of 0.2 ms duration, delivering TOF pulses of 2 Hz at 15-second intervals. The resulting contractions of the adductor pollicis muscle are quantified using the TOF-Watch SX. The acceleromyography is calibrated with 50 Hz tetanic stimulation for 5 seconds and TOF stimulation for 3 minutes. Next, muscle relaxation is induced using rocuronium bromide (0.6 mg/kg). Mechanical ventilation is initiated and the hypnotic depth is maintained with a bispectral index score (BIS) in the range of 45 to 60 using BIS VISTA (Aspect Medical Systems, Inc., Norwood, MA). A continuous infusion of rocuronium is used to maintain moderate (TOF count of 1 or 2) or deep (post-tetanic count [PTC] of 1 or 2) NMB. An additional rocuronium bolus of 0.15 mg/kg can be injected if the surgeon requires a greater depth of NMB to achieve an adequate surgical field. Sugammadex is injected to the deep NMB group and the moderate NMB group at 4 mg/kg and 2 mg/kg, respectively.

### 2.6 Operation procedure

A standard radical distal gastrectomy with D1 + or D2 lymphadenectomy is performed in both groups. The extent of lymphadenectomy is determined according to the Japanese Gastric Cancer Treatment Guidelines 2014. Partial omentectomy is performed and reconstruction is performed in the standard Billroth II (with or without Braun anastomosis), Roux-en-Y, or uncut Roux-en-Y fashion, depending on the preference of the surgeon. The intra-abdominal pressure during operation is 14 mmHg.

### 2.7 Outcome measurements

**Figure 2:**
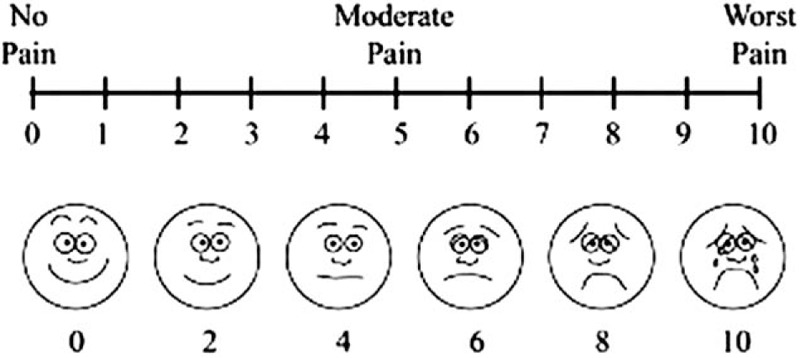
Visual analogue scale.

To measure the primary outcome, the number of harvested lymph nodes will be detected through a formal pathologic report. The anesthesiologists measure peak inspiratory pressure every 15 minutes, as well as total anesthesia time, total operation time, time from the end of the operation to the administration of the reversal agent, time from the administration of the reversal agent to the point at which the TOF ratio is greater than 0.9. They also check extubation time, the time to eye-opening to verbal commands, and the time to unassisted breathing. In the post-anesthesia care unit, the anesthesiologists monitor the sedation score (Table 1), and pain score using the visual analog scale (VAS) (Fig. 2). The number of episodes of nausea and vomiting as well as dosage and registered name of rescue analgesics and antiemetics are also measured.

**Table 1:**
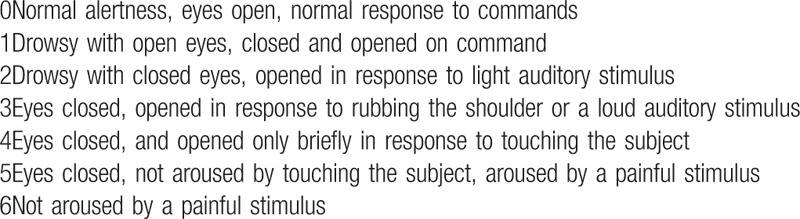
Sedation score.

During the operation, the surgeon reports the surgical rating score (Table 2) using Leiden surgical rating scale^[20]^ (Table 2) check 4 times during lymph node dissection (4sb, 6, supraduodenal, suprapancreatic area). They also note any problems associated with the operation, particularly in relation to muscle relaxation and symptoms associated with insufficient muscle relaxation, and the amount of intraoperative bleeding is measured.

**Table 2:**
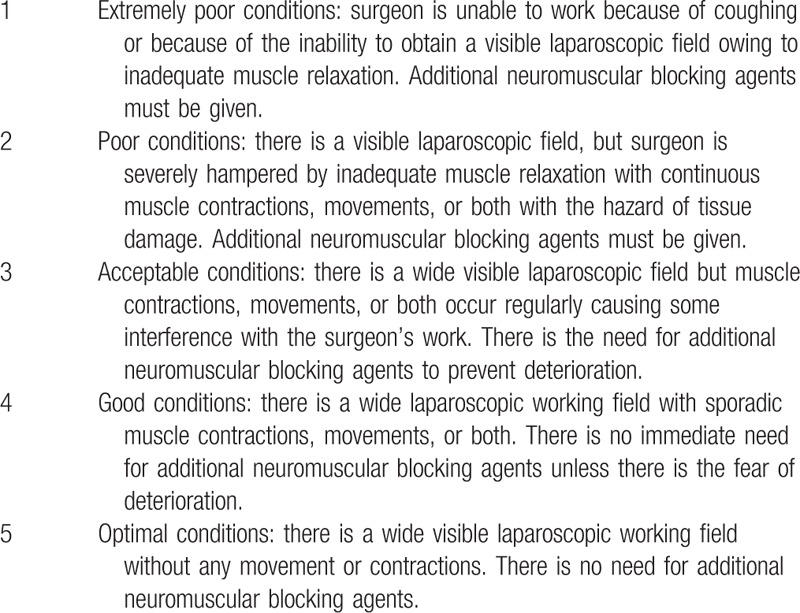
Surgical rating score.

After operation, the time to first flatus is monitored, and postoperative complications within 30 days are checked using the Clavien-Dindo classification of surgical complications.^[21]^

### 2.8 Sample size calculation

The hypothesis being tested is that the number of retrieved lymph nodes is greater in the deep NMB group than in the moderate NMB group. As there are no previous studies examining the oncological effects of cancer surgery with varying degree of NMB, we calculated the sample size based on a study comparing a low visceral fat area and a high visceral fat area.^[22]^ According to this study, the mean values of harvested lymph nodes for each group were 42.2 and 35.3, respectively. The common standard deviation was 13.5. We calculated that 82 patients for each group provide 90% power to detect a difference between the 2 groups using a 2-sided Student test with α of 0.05. The calculations are the customary ones based on normal distributions: Estimation of sample size and power for comparing 2 means in Bernard Rosner's Fundamentals of Biostatistics.^[23]^ 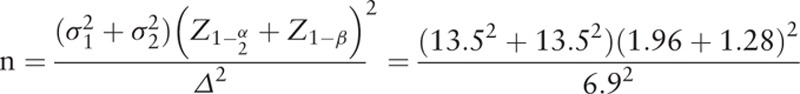


To account for potential patient dropouts (20%), the sample size is estimated at 196 patients (98 per group).

### 2.9 Data analysis plan

Statistical analysis will be conducted using SPSS software (SPSS version 20.0, IBM Corp., Chicago, IL). Quantitative variables will be expressed as means, and the Shapiro–Wilk test will be performed to test the normality of the variables. For normally distributed data, Student *t* test will be used, and the Mann–Whitney *U* test will be used for other data. Qualitative variables will be converted to numbers using score card, percentage, and 95% confidence intervals. The various parameters will be compared using Pearson and Fisher Chi-squared tests. For all the statistical tests, a *P* value less than .05 in a 2-tail test will be considered significant.

### 2.10 Management of adverse effects

Patients’ general medical conditions are examined throughout the study to monitor adverse effects. An adverse effect is defined as a change in physiologic or mental status resulting from the study. Severe adverse effects include the following: death, life threat, perpetual disability, and increment in hospitalization period, increment in drug use (greater than 25% increment over normal use). If a severe adverse effect or unexpected adverse effect assumed to be severe is identified, we will report the event to the IRB, and adjust the study methods.

### 2.11 Monitoring plan

Compliance of all participating investigators with the study protocol will be verified every month. In the case of a serious technical problem on the part of a surgeon that distorts the outcome in interim analysis, we would drop the surgeon from the study.

### 2.12 Ethics and dissemination plans

The study protocol was approved by the IRB of each participating institution. The results will be presented at national and international meetings. The results will be published in open-access peer-reviewed journals. All participating investigators can propose research suggestions and publish research papers based on the study data.

## 3 Discussion

This is the first study of a double-blind, randomized controlled multicenter prospective trial designed to examine the oncological benefits of deep NMB in obese patients with gastric cancer. Although laparoscopic gastrectomy has been widely adopted for early gastric cancer, obesity is a technical limiting factor during the laparoscopic procedure. Moreover, obesity is associated with postoperative complications. To predict the impact of obesity on surgical results, it is necessary to precisely estimate the extent of obesity. Many parameters including visceral fat area, abdominal shape, actual body fat area using CT, and BMI can be used to verify the extent of obesity. Some investigators have suggested that visceral fat area is a suitable parameter for obese patients with gastric cancer who undergo laparoscopy-assisted distal gastrectomy.^[24]^ Another study reported that abdominal shape can influence lymph node dissection during laparoscopic gastrectomy in gastric cancer patients.^[25]^ Among these parameters, BMI is a compelling alternative because it can be calculated with ease. Using BMI, several studies have shown worse surgical outcomes in obese patients with gastric cancer.^[26–28]^ There has been little research regarding how to safely perform laparoscopic gastrectomy in obese patients. Therefore, we propose the DEBLOQS_GC study.

The primary endpoint of this prospective study is the number of harvested lymph nodes. In gastric cancer, the guidelines recommend removal of 15 or more lymph nodes. Although the direct benefits of extended lymph node dissection are controversial,^[29]^ the number of harvested lymph nodes is an easily measurable indicator to evaluate the quality of gastric cancer surgery.

The relationship between BMI and the number of harvested lymph nodes during gastric cancer surgery is a controversial issue. One study reported a decreased number of harvested lymph nodes in patients with high BMI,^[30]^ whereas others found no relationship between lymph nodes and BMI.^[31,32]^ In contrast, another study reported an increased number of harvested lymph nodes in patients with high BMI.^[33]^ The reason for these results might be that there is a tendency for BMI to be less influenced by the development of surgical skills. Moreover, for the same BMI, the amount of intra-abdominal fat varies according to gender or race. However, it is undeniable that high BMI is a factor in unsuccessful lymph node dissection.

Until recently, the use of deep NMB was hindered by long recovery time and postoperative respiratory complications.^[34,35]^ However, the recent introduction of sugammadex has made reversal of deep NMB clinically feasible without postoperative complications.^[36]^ In contrast to other agents, sugammadex can bind to rocuronium in plasma, inducing rapid and intense reversal of muscle relaxation.^[37]^ Indeed, deep NMB became a general option with sugammadex.

The direct benefits of deep NMB for surgeons and patients have been debated.^[14,38]^ However, increasing numbers of studies have reported that deep NMB could provide improved surgical conditions and less pain compared to moderate NMB during laparoscopic surgery using sugammadex.^[39,40]^ The reasons for these results might be that deep NMB can provide a more favorable surgical field as the muscle contracts at a lower frequency. Moreover, intra-abdominal pressure can be lowered during surgery using deep NMB, which can be a factor in decreased postoperative pain.^[41,42]^

As mentioned previously, there have been no earlier studies comparing deep NMB with moderate NMB during laparoscopic gastrectomy in patients with gastric cancer. Moreover, to the best of our knowledge, there are no studies that examined the objective surgical effect of deep NMB over moderate NMB. Consequently, the DEBLOQS_GC study was designed to show the effect of deep NMB during laparoscopic gastrectomy in obese patients with gastric cancer. The results of this study will play an important role in identifying the optimal degree of muscle relaxation during laparoscopic gastrectomy in obese patients.

## 4 Declarations

### 4.1 Ethics approval and consent to participate

Patients consented to the anonymous use of their data for study purposes. The study has been approved by the IRB of Korea University Medical Center (IRB No. ED17059) and each participating institution: Asan Medical Center, Chonnam National University Hwasoon Hospital, Chung Ang University Hospital, Dongnam Institute of Radiological & Medical Science, Korea University Medical Center Anam Hospital, Korea University Medical Center Ansan Hospital, Korea University Medical Center Guro Hospital, Kyung Hee University Gangdong Hospital, and Kyungpook National University Chilgok Hospital. The study protocol was registered at clinicaltrial.gov (NCT03196791), date of registration October 10, 2017.

Before registration written informed consent will be obtained in all patients. Currently, we are recruiting patients in each participating institution.

### 4.2 Consent for publication

Not applicable.

### 4.3 Availability of data and materials

The datasets of the present study are available from the corresponding author on reasonable request.

### 4.4 Competing interests

The authors declare that they have no competing interests.

## Author contributions

HH and SP designed the protocol. DH, YL, and YJJ drafted the manuscript. All other authors participated in the design of the study. YL, LA, YJJ, and CML will analyze the data. HH, JHK, SHP, IOL, and YJM will interpret the data. All authors were involved in editing the manuscript and approved the final text of the manuscript. All authors read and approved the final manuscript

**Conceptualization:** You-Jin Jang, Sungsoo Park.

**Data curation:** Liang An.

**Formal analysis:** Chang Min Lee.

**Methodology:** Donghwan Ha, You-Jin Jang.

**Supervision:** Hyub Huh, Sungsoo Park.

**Writing – original draft:** Yoontaek Lee, Donghwan Ha.

**Writing – review & editing:** Hyub Huh, Yeon-Hee Kim, Jong-Han Kim, Seong-Heum Park, Young-Jae Mok, Il Ok Lee, Oh Kyoung Kwon, Kyung Hwa Kwak, Jae Seok Min, Eun Jin Kim, Sung Il Choi, Jae Woo Yi, Oh Jeong, Mi Ran Jung, Hong Bum Bae, Joong-Min Park, Yong Hoon Jung, Jin-Jo Kim, Dal Ah Kim, Sungsoo Park.
