# Comparison of oncological benefits of deep neuromuscular block in obese patients with gastric cancer (DEBLOQS_GC study): A study protocol for a double-blind, randomized controlled trial: Erratum

**DOI:** 10.1097/MD.0000000000014141

**Published:** 2019-01-04

In the article, “Comparison of oncological benefits of deep neuromuscular block in obese patients with gastric cancer (DEBLOQS_GC study): A study protocol for a double-blind, randomized controlled trial”,^[1]^ which appeared in Volume 97, Issue 49 of *Medicine*, the affiliations for Drs. Jin-Jo Kim and Dal Ah Kim appeared incorrectly. Dr. Jin-Jo Kim's affiliation should be Department of Surgery, Incheon St. Mary's Hospital, The Catholic University of Korea College of Medicine, Incheon, Korea. Dr. Dal Ah Kim's affiliation should be Department of Anesthesiology and Pain Medicine, Incheon St. Mary's Hospital, The Catholic University of Korea College of Medicine, Incheon, Korea.
